# Inferior Pulmonary Ligament Division May Be Unnecessary during Left Upper Lobectomy: Effects on Lung Volume, Bronchial Angle and Bronchial Tortuosity

**DOI:** 10.3390/jcm10184033

**Published:** 2021-09-07

**Authors:** Duk Hwan Moon, Chul Hwan Park, Joon Ho Jung, Tae Hoon Kim, Seok Jin Haam, Sungsoo Lee

**Affiliations:** 1 Department of Thoracic and Cardiovascular Surgery, Gangnam Severance Hospital, Yonsei University College of Medicine, Seoul 06273, Korea; PUPUPUCK@yuhs.ac (D.H.M.); alien31337@hanmail.net (J.H.J.); 2 Department of Radiology and Research Institute of Radiological Science, Gangnam Severance Hospital, Yonsei University College of Medicine, Seoul 06273, Korea; park_chulhwan@yuhs.ac (C.H.P.); thkim1@yuhs.ac (T.H.K.); 3 Department of Thoracic and Cardiovascular Surgery, Ajou University School of Medicine, Suwon 16499, Korea

**Keywords:** lobectomy, inferior pulmonary ligament, lung volume, bronchial angle, bronchial tortuosity, computed tomography

## Abstract

The benefits of dissecting inferior pulmonary ligament (IPL) during upper lobectomy using video-assisted thoracoscopic surgery (VATS) for early-stage lung cancer remains controversial. This study evaluates the effect of IPL dissection by comparing the lung volume, bronchial angle, and bronchial tortuosity of the left lower lobe (LLL) during VATS upper lobectomy. Medical records of all patients who underwent VATS left upper lobectomy for early-stage lung cancer were evaluated. Patients were divided into group P (preservation) and group D (dissection). Pre- and post-surgery lung volumes, bronchial angles (angle 1: axial angulation; angle 2: vertical angulation), and bronchial tortuosity (curvature index of the left main bronchus) were measured using computed tomography images for comparison. Forty patients were included in each group. Patient characteristics such as age, gender, body mass index, and smoking status, and preoperative lung volume, bronchial angles, and tortuosity were not significantly different between the two groups, and there was no statistically significant difference in the axial and vertical angulations; however, the change in pre- and postoperative bronchial tortuosity (0.03 ± 0.03 vs. 0.06 ± 0.03) and lung volume (−558.1 ± 410.0 mL vs. −736.3 ± 382.7 mL) showed a significant difference (*p <* 0.001 and *p =* 0.04, respectively). Preservation of IPLs during left upper lobectomy may be beneficial for LLL expansion and induces less movement and positional change in the left main bronchus.

## 1. Introduction

Several thoracic surgeons believe that performing inferior pulmonary ligament (IPL) dissection during upper lobectomy leads to positive postoperative outcomes with respect to lung expansion, reduction in dead space and its accompanying pleural effusion, reduced chest tube indwelling time, and prevention of atelectasis [1,2,3,4,5]. However, it has been reported that excessive lung expansion can cause severe change in the bronchial angulation that ultimately induces bronchial deformity [1,3,5].

In addition, some surgeons believe that IPL preservation may be beneficial in terms of postoperative pleural fluid drainage by providing normal lymphatic drainage and channel [6,7,8,9]. They also assert that IPL preservation may offer greater physiological benefits owing to the minimal change in the bronchial angle [3,4].

However, it is important to consider that IPL preservation may lead to insufficient lung expansion, causing pleural effusion and atelectasis [5,10,11]. Despite some studies in the literature, IPL preservation efficacy remains controversial. To the best of our knowledge, there is no convincing evidence to date suggesting whether dissection or preservation of IPL during upper lobectomy improves the postoperative outcomes and reduces the development of complication.

Therefore, this study attempted to analyze the impact of IPL dissection and/or preservation on the expansion, bronchial angle change, and bronchial tortuosity change of the left lower lobe after left upper lobectomy.

## 2. Materials and Methods

This study was approved by the ethics committees/institution review boards of both institutions (Gangnam Severance Hospital and Ajou University Hospital). The requirement for written informed consent was waived due to the retrospective nature of this study. All methods were performed in accordance with the relevant guidelines and regulations

### 2.1. Patients

**Figure 1:**
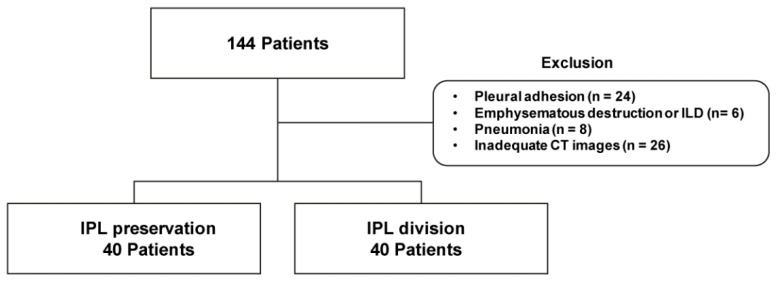
Flowchart of patient selection.

This retrospective study reviewed and analyzed the medical records of patients who underwent left upper lobectomy using VATS for early-stage cancer between January 2014 and January 2017 at two institutions. The National Health Insurance Service database was also retrospectively reviewed. Parameters such as demographic characteristics, operative details, and pathologic findings were evaluated. Lung cancer staging was determined using the eighth edition of the American Joint Committee on Cancer TNM staging system. Overall, 144 patients were initially included. Exclusion criteria were severe pleural adhesion discovered during the operation (*n* = 24), emphysematous destruction or interstitial lung disease (*n* = 6), cancer-related pneumonia on preoperative chest computed tomography (CT) (*n* = 8), and inadequate/unsuitable CT images (*n* = 26) (Figure 1). All included patients underwent CT scan within one month prior to the surgery and at postoperative six months.

### 2.2. Surgery

One surgeon from each institution performed surgeries for this study. Depending on the preference of each surgeon, IPL preservation was performed at one institution and IPL dissection in another. However, at both institutions, both surgeons adhered to the same oncological principle, performing complete anatomical lobectomy and mediastinal lymph node dissection. In all patients, double-lumen endotracheal intubation was used for administering general anesthesia, and single-lung ventilation was used for performing the surgery. Three incisions were made, and fissure-based technique was used for performing VATS lobectomy. Moreover, endoscopic staplers were used for the division of hilar structures, including the pulmonary vessels and bronchus. All patients were extubated after the surgery in the operating room. The chest tube was removed if there was no air leakage and when the drain volume was in the range between 200 and 250 mL/day based on the patient’s weight.

### 2.3. Chest CT Protocol

Chest CT scans were performed by various multi-detector CT machines including: Somatom Sensation 16, Somatom Sensation 64 and Somatom Definition AS+ (Siemens Medical Solutions, Erlangen, Germany). Participants were scanned from the lung apex to the adrenal glands in supine position during breath-holding at the end of inspiration. Volumetric CT scans were obtained from all patients with the following parameters: 120 kVp, 100–150 mAs, pitch of 1, 1.0 mm section thickness, and contiguous or overlapping section interval.

### 2.4. Chest CT Analysis—Lung Volume Measurement

**Figure 2:**
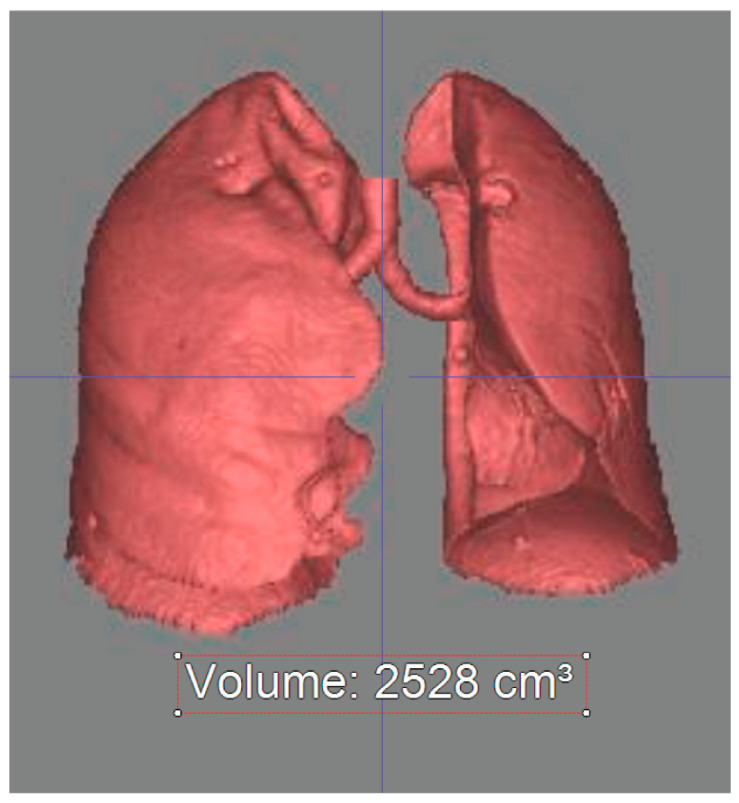
Three dimensional lung volume measurement on chest CT.

Three-dimensional lung volumes were semi-automatically measured using a commercially available reconstruction software (Aquarius iNtuition^™^ Ver.4.4.6; TeraRecon, Foster City, CA, USA) according to the previously reported threshold-based, three-dimensional auto-segmentation technique (Figure 2).

Anatomical lung volume was measured by using a density mask technique and auto-segmentation technique. Voxels with HU values ranging from −1024 HU to −200 were selected on the axial CT images and their volume was measured semi-automatically.

### 2.5. Chest CT Analysis—Bronchial Angle Measurement

**Figure 3:**
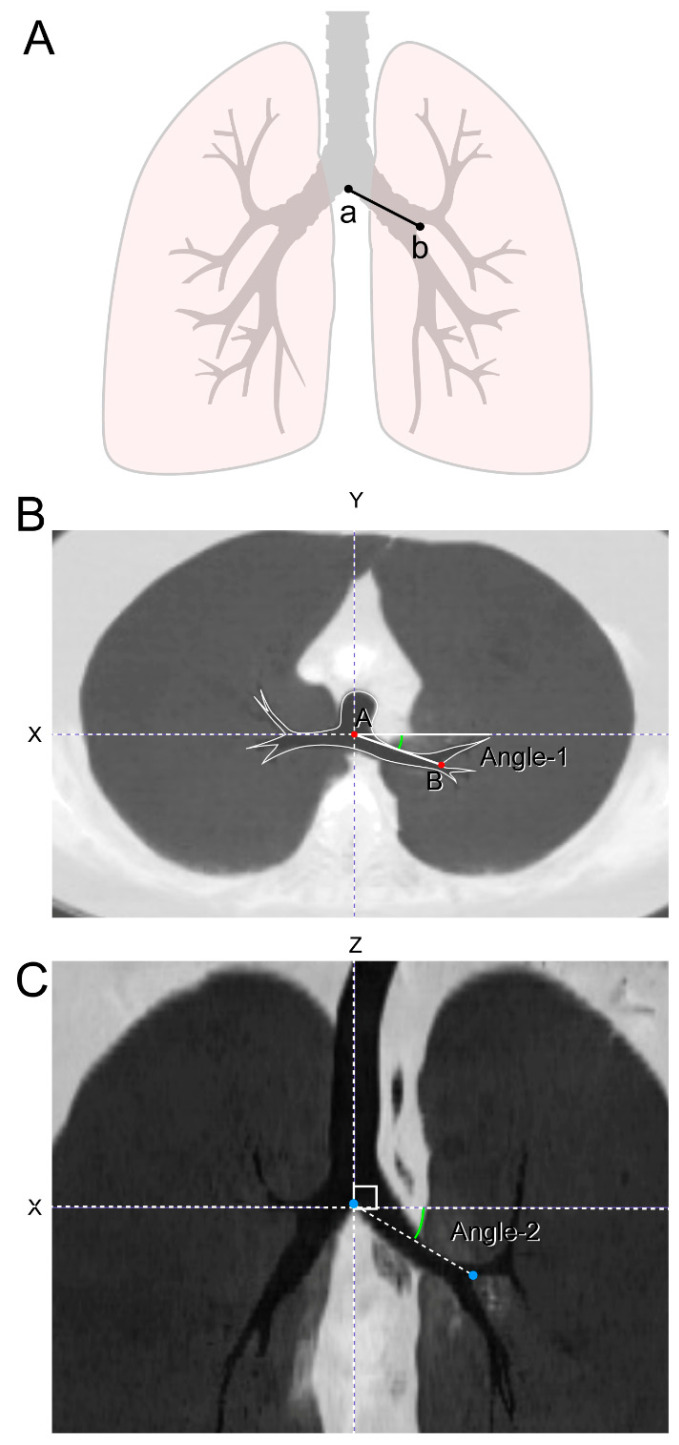
Measurement of bronchial angles. (**A**) The angulation of left main bronchus was measured in two ways as follows; (**B**) Angle 1—horizontal angulation: the angle of the left main bronchus formed on the horizontal plane when viewed from above. Y-axis—line passing through spinous process/vertebral body center and carina at the carina level. X-axis—line perpendicular to the Y-axis and passing through carina at the carina level. Angle 1 was measured as the angle formed between the X-axis and line through carina and second carina. If the angle was behind the X-axis, it was considered positive. If the angle was in front of the X-axis, it was considered negative. (**C**) Angle 2—vertical angulation: the angle of the left main bronchus formed on the vertical plane when viewed from the front. Z-axis—line passing through the carina and parallel to the trachea on the coronal plane. X-axis—line passing through the carina and perpendicular to the Z-axis on the coronal plane. Angle 2 was measured as the angle formed between the X-axis and line through carina and second carina. If the angle was below of the X-axis, it was considered positive. If the angle was above of the X-axis, it was considered negative.

On the axial images, the Y-axis was defined as a line intersecting the first carina and the midline of the vertebral body/spinous process. The X-axis was defined as an orthogonal line to the Y-axis across the first carina. On the coronal images, the Z-axis was defined as an orthogonal line to the XY plane across the carina and the midline of the trachea. The vertical angle of the left main bronchus was measured as the angle between the X-axis and the connecting line of the first and second carinas on the XZ plane. The angle returning to the inferior direction was expressed as a positive number. The horizontal angle of the left main bronchus was measured as the angle between the X-axis and the connecting line of the first and second carinas on the XY plane. The angle returned to the dorsal direction was expressed as a positive number (Figure 3).

### 2.6. Chest CT Analysis—Bronchial Tortuosity Measurement

**Figure 4:**
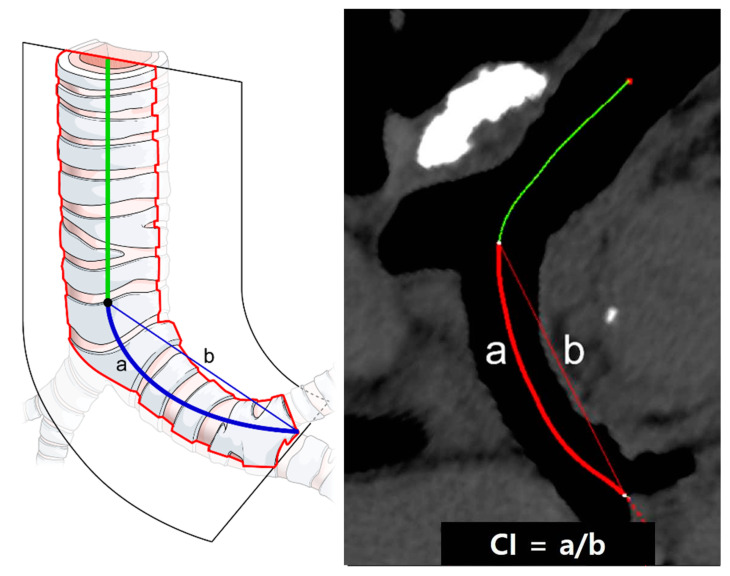
Measurement of left main bronchus tortuosity. Curvature index was used to measure the tortuosity of the left main bronchus. Center line passing through the distal trachea and left main bronchus was automatically created and carefully modified as needed. Distance of curved center line from carina to second carina (a) and over distance of straight line from carina to second carina (b) was used as curvature index (a/b), representing the tortuosity of left main bronchus. Higher indices indicated greater tortuosity.

Tortuosity of the left main bronchus was defined as the curvature index (CI): the curve length of the left main bronchus divided by the straight length of the left main bronchus. With commercially available reconstruction software, the full lengths of the left main bronchus were semi-automatically traced using the curved multiplanar technique; the CI was then calculated as the midline curve length of the left the main bronchus divided by the straight length using an electronic caliper after precise manual correction (Figure 4).

### 2.7. Statistical Analysis

Continuous variables were expressed as mean  ±  standard deviation and categorical variables as frequency or percentage. Normality of the variables was confirmed using the Shapiro–Wilk test. The following variables were compared between the two groups using the independent *t*-test: height, weight, and body mass index; the preoperative right lung volume, postoperative right lung volume, and pre- and post-operative right lung volume difference; the preoperative left lung volume, post-operative left lung volume, and pre- and post-operative left lung volume difference; the preoperative horizontal angle of the left main bronchus, post-operative horizontal angle of the left main bronchus, and pre- and post-operative horizontal angle difference; the preoperative vertical angle of the left main bronchus, post-operative vertical angle of the left main bronchus, and pre- and post-operative vertical angle difference; the preoperative CI of the left main bronchus, post-operative CI of the left main bronchus, and pre- and post-operative CI difference. The interobserver reproducibility for measurements (volume, angle, and CI) was assessed via intraclass correlation coefficients. A *p*-value of < 0.05 was considered statistically significant. All statistical analyses were performed with commercially available software SPSS 23 (IBM, Chicago, IL, USA).

## 3. Results

In this retrospective study, 80 patients were included; they were divided into two groups: 40 patients in the IPL preservation group (group P) and the remaining 40 patients in the IPL dissection group (group D). The average age in group P and group D was 63.6 ± 11.1 years and 64.2 ± 9.7 years, respectively, with no statistically significant difference. Moreover, there was no statistically significant difference between the two groups with respect to the number of men (%), height, weight, smoking status (%), chronic obstructive pulmonary disease (%), preoperative forced expiratory volume in 1 s (FEV1), preoperative forced vitality capacity (FVC), preoperative carbon monoxide lung diffusion capacity (DLCO), tumor size, and pathologic cancer stage. Regarding lung fissure during the operation, seven patients (17.5%) in group P and eight patients (20.0%) in group D had complete fissure, with no statistically significant difference. Chest tube indwelling time was 4.5 ± 3.8 days in group P and 4.8 ± 3.4 days in group D, with no statistically significant difference (Table 1).

### 3.1. Right Lung Volume

The preoperative right lung volume was 2409.8 ± 665.0 mL in group P and 2475.0 ± 500.0 mL in group D (*p =* 0.622). The postoperative right lung volume was 2619.1 ± 654.0 mL in group P and 2622.7 ± 505.8 mL in group D (*p =* 0.978). The difference between the preoperative and postoperative right lung volumes was 209.3 ± 454.2 mL in group P and 147.6 ± 344.1 mL in group D; there was no statistically significant difference between the two groups (*p =* 0.496) (Table 2).

### 3.2. Left Lung Volume

The preoperative left lung volume was 1970.9 ± 574.3 mL in group P and 2061.9 ± 515.3 mL in group D (*p =* 0.458). The postoperative left lung volume was 1412.8 ± 420.6 mL in group P and 1325.6 ± 383.3 mL in group D (*p =* 0.335). The difference between preoperative and postoperative left lung volume was −558.1 ± 410.0 mL (decrease of 28.3%) in group P and −736.3 ± 382.7 mL (decrease of 35.7%) in group D, which was statistically significant (*p* = 0.043) (Table 2).

### 3.3. Left Bronchial Angles

The preoperative angle 1 (axial angulation) was 13.5 ± 5.1° in group P and 14.3 ± 4.6° in group D (*p =* 0.497). The postoperative angle 1 was 10.6 ± 5.7° in group P and 8.5 ± 8.2° in group D (*p =* 0.198). The difference between the pre- and postoperative angle 1 was −2.9 ± 5.5° in group P and −5.7 ± 7.7° in group D. Although the difference was lower in group P, the difference was not statistically significant (*p =* 0.065). The preoperative angle 2 (vertical angulation) was 27.9 ± 5.5° in group P and 28.5 ± 6.9° in group D (*p =* 0.689). The postoperative angle 2 was 12.2 ± 9.0° in group P and 9.3 ± 9.8° in group D (*p =* 0.170). The difference between the pre- and postoperative angle 2 was −15.7 ± 8.4° in group P and −19.2 ± 11.0° in group D, with no statistically significant difference (*p =* 0.114) (Table 3).

### 3.4. Left Bronchial Tortuosity

The preoperative left main bronchus curvature index (CI) was 1.05 ± 0.02 in group P and 1.04 ± 0.02 in group D (*p =* 0.142). The postoperative left main bronchus CI was 1.08 ± 0.03 in group P and 1.11 ± 0.05 in group D (*p =* 0.003). The difference between pre- and postoperative left main bronchus CIs was 0.03 ± 0.03 in group P and 0.06 ± 0.03 in group D, with statistically significant difference (*p* < 0.001) (Table 4).

## 4. Discussion

According to our findings, the degree of left lung volume loss was less in group P than in group D, i.e., the expansion of the remaining lung was greater in the IPL preservation group. Although there was no statistically significant change in the postoperative bronchial angle, there was less variation in the postoperative bronchial angle in group P. Moreover, for the postoperative bronchial tortuosity, there was statistically significantly less bending in group P than in group D. The IPL maintains the structural integrity of the bronchus by minimizing the risk of torsion and kicking by fixating the residual (left lower) lung. We believe that this may play a role in residual lung expansion. 

According to several previous studies, the benefits of IPL dissection during upper lobectomy may be to enhance the range of motion of the inferior lobe and to improve the re-expansion of the inferior lobe, ultimately reducing postoperative complications, including atelectasis and pleural effusion, by improving the residual lung capacity of the superior lobe [1,2,4,5]. However, other studies failed to observe such benefits. Ueda et al. [12] reported that there was an increased frequency of bronchial distortion and stenosis in patients undergoing upper lobectomy with IPL dissection, leading to chronic cough, shortness of breath, etc. and ultimately resulting in reduced pulmonary function. Seok et al. [3] also observed that IPL dissection after upper lobectomy was associated with a greater frequency of bronchial distortion, resulting in poorer postoperative recovery of pulmonary function. Hence, to date, the benefits of IPL preservation remain controversial. 

In our study, a quantitative comparison was attempted between IPL preservation and IPL dissection during upper lobectomy; this was achieved by developing a bronchial tortuosity index. This newly developed index was devised to overcome the existing ambiguous method of bronchial angle measurement and unclear absolute reference when determining the angulation change. The bronchial tortuosity index appears to provide greater objectivity in measuring the bronchial angle and angulation change. Using this method, the physiological anatomy was better preserved (less change to the shape of the bronchus) in the IPL preservation group than in the IPL dissection group. We believe that IPL preservation promotes a positive impact on postoperative outcome, including reduced cough and improved pulmonary function. We also believe that IPL preservation allows for the maintenance of bronchial structure integrity, as indicated by the bronchial tortuosity index, and thereby positively influences postoperative lung volume. 

In our study, patients who underwent video-assisted thoracoscopic surgery (VATS) lobectomy were only included and those who underwent open thoracotomy lobectomy were excluded because, when compared with open thoracotomy, VATS tends to result in less pain and minimal alteration to the chest wall shape, thus having a relatively less negative impact on pulmonary function than open thoracotomy [13,14,15]. Moreover, patients with pleural adhesion were excluded because they would need to undergo adhesiolysis during surgery, which could lead to lung injury and the development of postoperative atelectasis or pleural effusion, ultimately resulting in physiological and anatomical alteration [16]. 

Advocates of IPL dissection when performing upper lobectomy consider the oncological aspects of IPL preservation as a disadvantage, i.e., they assert that without IPL dissection, there cannot be a clear dissection of the inferior mediastinal nodes, specifically the paraesophageal lymph node or the pulmonary ligament lymph node of the mediastinal lymph node in the lower zone [6,7,9]. IPL includes a few small bronchial veins, lymphatics, lymph nodes, etc. and may receive drainage from the basilar segments of the lower lobe [7,9]. As shown by Asamura et al. [17], mediastinal lymph node metastasis of the lower zone does not occur in upper lobe lung cancer. Moreover, a previous study showed that selective mediastinal lymph node dissection had no impact on survival [18]. Our study focused on patients in the early stages of lung cancer; thus, oncological issues may not be relevant for the purpose of this study.

There are several limitations to this study. First, this was a retrospective study with a small sample size. Second, two surgeons from two institutions—one surgeon per institution—performed surgeries, which may have impacted the results owing to factors based on the surgeon. However, the surgical techniques utilized are very similar between the two institutions, because surgeons from both of these institutions were trained at the same institution and there are highly shared activities. Third, the postoperative pulmonary function test was not performed in all patients on a regular basis; even if it was performed, we believe that it would have been difficult to compare the results between the two institutions. Moreover, postoperative evaluation and comparison of clinical symptoms were not performed because evaluating clinical symptoms via medical records review would have been inaccurate. Therefore, the results of this study may not provide an exact clinical implication. Fourth, we only included patients undergoing left upper lobectomy. This is because the right side of the lung is more complex than the left side, which would make IPL factors, including lung volume, bronchial angulation, and tortuosity, insufficient for analysis. However, our study may be noteworthy in that it attempted to present a newly developed parameter—bronchial tortuosity index—for evaluating the benefits of IPL preservation.

## 5. Conclusions

**Figure 5:**
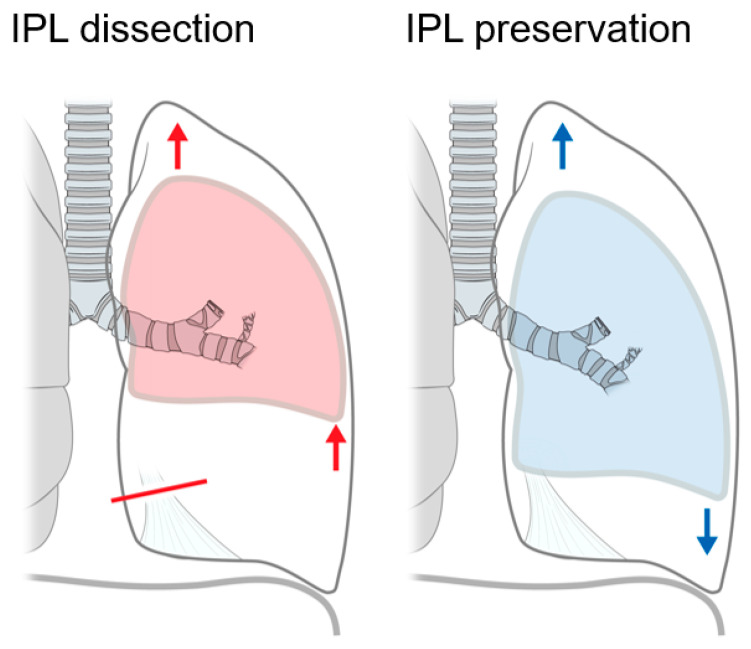
Effects of inferior pulmonary ligament preservation during left upper lobectomy. Red line: dissection of inferior pulmonary ligament (IPL); Red arrows: possible lung expansion with IPL dissection; Blue arrows: possible lung expansion with IPL preservation.

IPL preservation during left upper lobectomy may be beneficial for left lower lobe expansion and induces less movement and positional change to the left main bronchus (Figure 5). Nonetheless, larger future randomized studies are warranted for better evaluating the clinical benefits of IPL preservation.

Inferior pulmonary ligament preservation during left upper lobectomy may be beneficial for left lower lobe expansion with less movement and positional change of the left main bronchus.

## Figures and Tables

**Table 1:** Baseline characteristics between two groups.

Variables	Group P, *n* = 40	Group D, *n* = 40	*p*-Value
Age, years	63.6 ± 11.1	64.2 ± 9.7	0.825
Male, *n* (%)	24 (60%)	26 (65%)	0.817
Height, cm	162.9 ± 7.8	163.7 ± 8.2	0.677
Weight, kg	65.0 ± 8.4	64.7 ± 8.9	0.646
Smoking, *n* (%)	20 (50%)	24 (60%)	0.347
COPD, *n* (%)	0	2 (5%)	0.494
Preoperative FEV1, mL	1.98 ± 0.56	1.89 ± 0.48	0.598
Preoperative FVC, mL	2.73 ± 0.78	2.67 ± 0.64	0.669
Tumor size, cm	2.8 ± 1.9	2.7 ± 1.7	0.623
Histologic diagnosis, *n* (%)			0.785
Adenocarcinoma	34 (85%)	32 (80%)	
Squamous cell carcinoma	5 (12.5%)	6 (15%)	
Others	1 (2.5%)	2 (5%)	
Pathologic cancer stage, *n* (%)			0.485
I	27 (67.5%)	24 (60%)	
II	13 (32.5%)	16 (40%)	
Complete fissure, *n* (%)	7 (17.5%)	8 (20%)	0.980
CTD indwelling time, *n* (%)	4.5 ± 3.8	4.8 ± 3.4	0.598

COPD: chronic obstructive pulmonary disease; FEV1: forced expiratory volume in one second; FVC: forced vital capacity; CTD: chest tube drainage; P: preservation; D: division.

**Table 2:** The change in right and left lung volume between two groups.

	Group P, *n* = 40	Group D, *n* = 40	*p*-Value
**Right lung**			
Preoperative volume, mL	2409.8 ± 665.0	2475.0 ± 500.0	0.622
Postoperative volume, mL	2619.1 ± 654.0	2622.7 ± 505.8	0.978
Volume difference, mL	209.3 ± 454.2	147.6 ± 344.1	0.496
**Left lung**			
Preoperative volume, mL	1970.9 ± 574.3	2061.9 ± 513.3	0.458
Postoperative volume, mL	1412.8 ± 420.6	1325.6 ± 383.3	0.335
Volume difference, mL	−558.1 ± 410.0	−736.3 ± 382.7	0.043

P: preservation; D: division.

**Table 3:** The change in left bronchial angles between two groups.

	Group P, *n* = 40	Group D, *n* = 40	*p*-Value
**Angle 1**			
Preoperative angle 1,	13.5 ± 5.1	14.3 ± 4.6	0.497
Postoperative angle 1,	10.6 ± 5.7	8.5 ± 8.2	0.198
Angle 1 difference,	−2.9 ± 5.5	−5.7 ± 7.7	0.065
**Angle 2**			
Preoperative angle 2,	27.9 ± 5.5	28.5 ± 6.9	0.689
Postoperative angle 2,	12.2 ± 9.0	9.3 ± 9.8	0.170
Angle 2 difference,	−15.7 ± 8.4	−19.2 ±11.0	0.114

P: preservation; D: division.

**Table 4:** The change in left bronchial tortuosity between two groups.

	Group P, *n* = 40	Group D, *n* = 40	*p*-Value
CI			
Preoperative CI	1.05 ± 0.02	1.04 ± 0.02	0.142
Postoperative CI	1.08 ± 0.03	1.11 ± 0.05	0.003
CI difference	0.03 ± 0.03	0.06 ± 0.03	<0.001

CI: curvature index; P: preservation; D: division.

## Data Availability

The data presented this study are available on request from the corresponding author.

## References

[1] Usuda K., Sagawa M., Aikawa H., Tanaka M., Machida Y., Ueno M., Sakuma T. (2010). Do japanese thoracic surgeons think that dissection of the pulmonary ligament is necessary after an upper lobectomy?. Surg. Today.

[2] Khanbhai M., Dunning J., Yap K.H., Rammohan K.S. (2013). Dissection of the pulmonary ligament during upper lobectomy: Is it necessary?. Interact. Cardiovasc. Thorac. Surg..

[3] Seok Y., Yi E., Cho S., Jheon S., Kim K. (2015). Perioperative outcomes of upper lobectomy according to preservation or division of the inferior pulmonary ligament. J. Thorac. Dis..

[4] Bu L., Yang A.-R., Peng H., Xu Z.-Y., Wu J.-Q., Wang P. (2015). Dividing inferior pulmonary ligament may change the bronchial angle. J. Surg. Res..

[5] Kim D.H., Moon D.H., Kim H.R., Lee S.M., Chae E.J., Choi C.-M., Choi S.H., Kim Y.-H., Kim D.K., Park S.-I. (2019). Effect of inferior pulmonary ligament division on residual lung volume and function after a right upper lobectomy. Interact. Cardiovasc. Thorac. Surg..

[6] Clark R.A., Colley D.P. (1980). Pulmonary lymphatics visualized during pedal lymphangiography. Radiology.

[7] Cross G., Woodring J.H. (1987). Inferior pulmonary ligament lymphadenopathy: Demonstration by computed tomography. J. Comput. Tomogr..

[8] Murakami G., Adachi N., Sato I., Sato T., Hoshi H. (1994). Venous Drainage of the Thoracic Esophagus toward the Pulmonary Vein. Okajimas Folia Anat. Jpn..

[9] Okiemy G., Foucault C., Avisse C., Hidden G., Riquet M. (2003). Lymphatic drainage of the diaphragmatic pleura to the peritracheobronchial lymph nodes. Surg. Radiol. Anat..

[10] Rost R.C., Proto A.V. (1983). Inferior pulmonary ligament: Computed tomographic appearance. Radiology.

[11] Paling M.R., Griffin G.K. (1985). Lower lobe collapse due to pleural effusion: A CT analysis. J. Comput. Assist. Tomogr..

[12] Ueda K., Tanaka T., Hayashi M., Tanaka N., Li T.-S., Hamano K. (2012). Clinical Ramifications of Bronchial Kink After Upper Lobectomy. Ann. Thorac. Surg..

[13] Tajiri M., Maehara T., Nakayama H., Sakamoto K. (2007). Decreased invasiveness via two methods of thoracoscopic lobectomy for lung cancer, compared with open thoracotomy. Respirology.

[14] Paul S., Altorki N.K., Sheng S., Lee P.C., Harpole D.H., Onaitis M.W., Stiles B.M., Port J.L., D’Amico T.A. (2010). Thoracoscopic lobectomy is associated with lower morbidity than open lobectomy: A propensity-matched analysis from the STS database. J. Thorac. Cardiovasc. Surg..

[15] Yang D., Zhou Y., Wang W. (2018). Total thoracoscopic high-position sleeve lobectomy of the right upper lobe of the lung. J. Thorac. Dis..

[16] Rivera C., Bernard A., Falcoz P.-E., Thomas P., Schmidt A., Bénard S., Vicaut E., Dahan M. (2011). Characterization and Prediction of Prolonged Air Leak After Pulmonary Resection: A Nationwide Study Setting Up the Index of Prolonged Air Leak. Ann. Thorac. Surg..

[17] Asamura H., Nakayama H., Kondo H., Tsuchiya R., Naruke T. (1999). Lobe-specific extent of systematic lymph node dissection for non–small cell lung carcinomas according to a retrospective study of metastasis and prognosis. J. Thorac. Cardiovasc. Surg..

[18] Ishiguro F., Matsuo K., Fukui T., Mori S., Hatooka S., Mitsudomi T. (2010). Effect of selective lymph node dissection based on patterns of lobe-specific lymph node metastases on patient outcome in patients with resectable non–small cell lung cancer: A large-scale retrospective cohort study applying a propensity score. J. Thorac. Cardiovasc. Surg..

